# THE INTRINSIC CAPACITY PROFILE OF THE CHINESE OLDER ADULTS IN HONG KONG

**DOI:** 10.1093/geroni/igae098.0544

**Published:** 2024-12-31

**Authors:** Xiao-han Jiang, Feng Chen, Yiting Tang, Shuangzhou Chen, Doris Sau Fung Yu

**Affiliations:** The Hong Kong University, Guanzhou City, Guangdong, China (People’s Republic); The University of Hong Kong, Hong Kong, China (People’s Republic); The University of Hong Kong, Hong Kong, China (People’s Republic); The University of Hong Kong, Hong Kong, China (People’s Republic); The University of Hong Kong, Hong Kong, China (People’s Republic)

## Abstract

Intrinsic capacity (IC) refers to the composite of physical and mental capacity. To promote healthy ageing, the identification of IC will provide a clear picture of older adults. The IC assessment in “Pathway to Healthy Ageing Project” in Hong Kong was then conducted under the Integrated Care of Older People (ICOPE) framework. The 6 IC domains (cognition, locomotion, vitality, psychology, vision, and hearing) were assessed among the 4418 older adults recruited over Hong Kong (mean age: 74.4 years). Cognitive function was tested by Montreal cognitive assessment 5-min protocol. 16.6% of the participants had cognitive impairment. For locomotion, Short physical performance battery and Timed-Up and Go Test (TUG) were used to measure physical function and mobility. 35.0% were found to have impaired physical function, and 16.9% faced a fall risk. TUG revealed 6.5% of our participants had problem in mobility. Vitality was measured by Mini nutritional assessment and handgrip strength. Our results indicated that 27% of the participants had a malnutrition risk, and 0.4% were malnutrition. Handgrip strength indicated 59.4% had low muscle strength. Psychology was measured by Geriatric Depression Scale-15 item for depression. 10.8% of the participants were found with depression. Sensory was measured by self-reported. 22% reported they had visual loss, and 17.4% reported they had hearing loss. Our study provided a profile of IC of the community-dwelling older adults in Hong Kong. The IC impairment of Hong Kong older adults was identified, thus more targeted intervention can be provided to improve the overall health condition.

